# Association of increased renal *Cyp24a1* gene expression with low plasma 1,25-dihydroxyvitamin D levels in rats with streptozotocin-induced diabetes

**DOI:** 10.3164/jcbn.19-79

**Published:** 2019-10-30

**Authors:** Mari Tajiri, Otoki Nakahashi, Tomohiro Kagawa, Masashi Masuda, Hirokazu Ohminami, Masayuki Iwano, Eiji Takeda, Yutaka Taketani, Hironori Yamamoto

**Affiliations:** 1 Department of Clinical Nutrition and Food Management, Institute of Biomedical Sciences, University of Tokushima Graduate School, 3-18-15 Kuramoto-cho, Tokushima 770-8503, Japan; 2 Division of Functional Food Chemistry, Institute for Health Science, Tokushima Bunri University, 180 Nishihamahoji, Yamashiro-cho, Tokushima 770-8514, Japan; 3 Department of Nephrology, Faculty of Medical Sciences, University of Fukui, 23-3 Matsuoka, Shimoaizuki, Eiheiji-cho, Fukui 910-1193, Japan; 4 Department of Health and Nutrition, Faculty of Human Life, Jin-ai University, 3-1-1 Ohde-cho, Echizen-city, Fukui 915-8586, Japan

**Keywords:** vitamin D, CYP24A1, CYP24A1-SV, diabetes, streptozotocin

## Abstract

Decreases in plasma vitamin D concentrations have been reported in diabetes, although the mechanism involved in this decrease is unclear. Here, we investigated the association between Cyp24a1, a vitamin D catabolic enzyme, and abnormalities in vitamin D metabolism in streptozotocin-induced diabetes rats, an animal model of type 1 diabetes. Plasma 1,25-dihydroxyvitamin D [1,25(OH)_2_D] levels were significantly lower in streptozotocin-induced diabetes rats and renal *Cyp24a1* mRNA expression levels were increased. Western blotting analysis of streptozotocin-induced diabetes rats kidney tissues with anti-CYP24A1 antibody showed a strong signal around 40 kDa, which differs from the predicted 50–55 kDa molecular weight for full-length Cyp24a1 and could represent the Cyp24a1-splicing variant that lacks exons 1 and 2. We observed high levels of renal Cyp24a1-splicing variant mRNA expression in streptozotocin-induced diabetes rats. We also confirmed transcriptional up-regulation of endogenous *Cyp24a1* mRNA expression through glucocorticoid receptors by glucocorticoid in opossum kidney proximal cells. Taken together, our results indicated that high *Cyp24a1* expression levels may play a role in the decrease of plasma 1,25(OH)_2_D levels in streptozotocin-induced diabetes rats. High plasma corticosterone levels in diabetes may affect transcriptional regulation to promote increases in *Cyp24a1* expression.

## Introduction

Vitamin D, absorbed from the diet or synthesized from 7-dehydrocholesterol in the skin upon exposure to solar ultraviolet B (UVB), is metabolized in the liver to 25-dihydroxyvitamin D_3_ [25(OH)D_3_], which is the major circulating form of vitamin D.^(1)^ 25(OH)D_3_ is metabolized to 1,25-dihydroxyvitamin D_3_ [1,25(OH)_2_D_3_] by the mitochondrial cytochrome P450 enzyme 25-hydroxyvitamin D_3_-1α-hydroxylase (encoded by *CYP27B1*) in the kidney.^(1)^ The biologically active form of vitamin D, 1,25(OH)_2_D_3_, regulates calcium (Ca) and inorganic phosphate (Pi) homeostasis, as well as bone remodeling. 1,25(OH)_2_D_3_ binds to vitamin D receptor (VDR) and regulates expression of several genes, including transient receptor potential (TRP) cation channel subfamily V members 5 and 6 (TRPV5 and TRPV6), which are the molecular gatekeepers that facilitate Ca^2+^ influx in the kidney and the duodenum, respectively.^(1,2)^ 1,25(OH)_2_D_3_ induces TRPV5 and TRPV6 expressions. Both 1,25(OH)_2_D_3_ and its precursor 25(OH)D_3_ are metabolized in the kidney by the mitochondrial cytochrome P450 enzyme 25-dihydroxyvitamin D_3_-24-hydroxylase, encoded by the gene *CYP24A1*, and catabolized to 1,24,25(OH)_3_D_3_ and to 24,25(OH)_2_D_3_, respectively, the biologically inactive forms of vitamin D.^(1)^ In the kidney, genes involved in vitamin D synthesis such as *CYP27B1* as well as vitamin D catabolic enzymes including *CYP24A1* are mainly expressed in renal proximal tubular cells and determine circulating levels of 1,25(OH)_2_D_3_.^(2)^ Expression of *CYP27B1* and *CYP24A1* are tightly regulated by parathyroid hormone (PTH), fibroblast growth factor 23 (FGF23), and 1,25(OH)_2_D_3_. *CYP27B1* expression is induced by PTH, and inhibited by FGF23 and 1,25(OH)_2_D_3_.^(1)^ In contrast, *CYP24A1* expression is induced by FGF23 and 1,25(OH)_2_D_3_,^(1)^ and inhibited by PTH.^(3)^ Vitamin D metabolism is also regulated by factors associated with glucose or energy metabolism, including insulin,^(2)^ insulin-like growth factor 1 (IGF1),^(4)^ leptin,^(5)^ and glucocorticoid.^(6)^

Activities of mitochondrial cytochrome P450 enzymes, including CYP27B1 and CYP24A1, are dependent on the NADPH-adrenodoxin-reductase electron transport system that localizes to mitochondria.^(7)^ Ren *et al.*^(8)^ first reported that the *CYP24A1*-splicing variant (*CYP24A1-SV*), which is spliced at intron 2 and lacks exons 1 and 2, occurs during transcription of the human and chick *CYP24A1* gene. They suggested that the CYP24A1-SV protein retains the substrate-binding domain, but lacks the N-terminal mitochondrial targeting domain encoded by exon 1 of *CYP24A1*. As such, CYP24A1-SV may have a cytosolic localization and is functionally inactive.

The coexistence of diabetes and vitamin D metabolic disorder has been established by a number of investigations involving diabetic patients. Insulin-dependent type 1 diabetes patients consistently have low vitamin D levels and even lower serum concentrations of 25(OH)D_3_^(9,10)^ and/or 1,25(OH)_2_D_3_.^(11)^ Type 2 diabetes patients also have low serum 25(OH)D_3_ levels,^(12)^ while woman with metabolic syndrome have high serum 25(OH)D levels.^(13)^ In the *db/db* mouse model of type 2 diabetes, up-regulation of renal *Cyp24a1* expression is associated with decreased vitamin D levels.^(14)^ Moreover, Vuica *et al.*^(15)^ showed a significant increase in *Cyp24a1* expression in hepatocytes from long-term type 1 diabetes rats induced by streptozotocin (STZ), which can injure pancreatic β cells. However, the CYP24A1 enzyme is expressed mainly in the kidney and at only very low levels in the liver.^(16)^ In contrast to the increased expression levels in the liver, Zhang *et al.*^(17)^ reported that renal *Cyp24a1* expression is inhibited in type 1 diabetic mice injected with STZ. As such, there is an incomplete understanding of the association between *Cyp24a1* expression and the low vitamin D levels in diabetes, and levels of 1,25(OH)_2_D_3_ in rats having STZ-induced type 1 diabetes have not been measured before. In the present study, we investigated the relationship between changes in Cyp24a1 expression and abnormalities in vitamin D metabolism in STZ-induced diabetic rats.

## Materials and Methods

### Animals

Five-week-old male Sprague-Dawley (SD) rats (Japan SLC, Hamamatsu, Japan) were kept on a 12 h light/12 h dark cycle at constant temperature. To induce type 1 diabetes, rats were intraperitoneally (ip) injected with 65 mg/kg B.W. STZ (Wako, Osaka, Japan) in citrate buffer (pH 4.5) (STZ rats), or citrate buffer alone ip (control rats). Some STZ rats were treated subcutaneously (sc) with 2 U insulin (Humulin N insulin, Lilly, Indianapolis, IN) twice daily from Day 4 to Day 9 after STZ injection (STZ + Insulin rats). Rats were fed a diet including calcium 0.6% and phosphate 0.6%, and allowed free access to the diet and water. On Day 9 after STZ injection, the rats were sacrificed. Body weight and food intake were measured daily. Protocols for breeding and handling as well as the experimental protocols for all experiments involving animals were approved by the Animal Experimentation Committee of Tokushima University.

### Blood parameters

Concentrations of insulin and glucose levels in blood samples were determined using the Ultra Sensitive Rat Insulin Kit (MORINAGA, Kanazawa, Japan) and LabAssay^TM^ Glucose, respectively. Blood urea nitrogen (BUN), creatinine, Ca, and Pi were measured using Urea Nitrogen B test, Creatinine test, Calcium E-test, and Phospha C-test, respectively (all from Wako). Concentrations of corticosterone, PTH, FGF23, osteocalcin, and tartrate-resistant acid phosphatase-5b (Trap-5b) were determined using YK240 Corticosterone EIA (Yanaihara Institute Inc., Shizuoka, Japan), Rat Intact PTH ELISA Kit (Immutopics, San Clemente, CA), FGF23 ELISA Kit (Kinos, Tokyo, Japan), Rat Osteocalcin ELISA Kit DS (DS PHARMA, Osaka, Japan), and EIA Rat TRAP-5b (Nittobo Medical, Tokyo, Japan), respectively. Plasma 1,25(OH)_2_D levels were measured with a radioimmunoassay (RIA) kit (TFB, Tokyo, Japan) and plasma 25(OH)D levels were measured with a RIA method (DiaSorin, Stillwater, MN). Plasma collected with heparin from the abdominal aorta was used for all tests.

### RT-PCR analysis and quantitative real-time PCR analysis

Total RNA from the kidney, duodenum, and cells was extracted using RNA iso plus reagent (Takara Bio, Shiga, Japan), and then dissolved in RNase-free water. First-standard cDNA was synthesized from 2.5 µg total RNA primed with oligo (dT) using M-MLV-reverse transcriptase (Invitrogen, Carlsbad, CA).

RT-PCR was performed using PCR thermocycler (Eppendorf, Hamburg, Germany). The amplification program was 95°C for 1 min, followed by 27 cycles for *Cyp24a1* and *Cyp27b1* or 20 cycles for *β-actin* that were: 95°C for 45 s, 58°C for 45 s and 72°C for 60 s. The prepared first-strand cDNA was PCR amplified using Master Mix (Promega, Madison, WI) in a 20 µl reaction volume, with 4 pmol of each primer (Table 1). *β-actin* was used as an internal control.

Quantitative real-time PCR was performed using the StepOnePlus^TM^ Real-Time PCR System (Applied Biosystems, Foster City, CA). The amplification program was 95°C for 10 min, and then 40 cycles of 95°C for 10 s, 60°C for 15 s and 72°C for 15 s. The prepared first-strand cDNA was PCR-amplified using Fast SYBR^®^ Green Master Mix (Applied Biosystems) in a 20 µl reaction volume, with 4 pmol of each primer (Table 1). The amplification products were then analyzed using a melting curve, which confirmed the presence of a single PCR product in all reactions (except for the negative controls). The PCR products were quantified by fit-point analysis, and the results were normalized to those for *β-actin*. The gene-specific paired primer sequences are shown in Table 1.

### Protein extractions

Opossum kidney proximal (OK-P) cells were homogenized in TNE Buffer (10 mM Tris-HCl, pH 7.8, 1% Nonidet P-40, 150 mM NaCl, 1 mM EDTA with trypsin inhibitor, Pepstatin A, DTT, PMSF), and centrifuged at 10,000 rpm for 15 min at 4°C after incubation on ice for 30 min. The supernatants contain whole cell proteins from OK-P cells.

Kidneys were homogenized in lysis buffer (50 mM Tris-HCl, pH 7.5, 1% Triron X-100, 150 mM NaCl, 5 mM EDTA) containing protease inhibitor, kept for 30 min on ice, and centrifuged at 12,000 rpm for 20 min at 4°C to yield supernatants carrying whole cell proteins of kidney tissues.

Kidneys were also homogenized in CP-1 buffer (50 mM Tris-HCl, pH 7.5, 100 mM KCl, 150 mM NaCl, 2 mM EGTA, 5 mM EDTA) containing protease inhibitor, and centrifuged at 500 G for 10 min at 4°C. The pellets were resuspended in CP-1 buffer and centrifuged at 2,000 rpm for 5 min at 4°C. The resulting pellets were then resuspended in hypotonic buffer, kept on ice for 10 min, and centrifuged at 3,000 rpm for 4 min at 4°C. These pellets were dissolved in extraction buffer, kept on ice for 1 h, and centrifuged at 15,000 rpm for 45 min at 4°C. The supernatants after this fourth centrifugation contained the nuclear fraction. The supernatants obtained after the first centrifugation were filtered with cell strainers (BD Falcon REF352340 40 nm, BD, Tokyo, Japan), centrifuged at 10,500 *g* for 10 min at 4°C and concentrated with Amicon Ultra filter units (Millipore, Bedford, MA) to yield cytosolic fraction. The pellets obtained after the second centrifugation were dissolved in RIPA buffer containing protease inhibitor, kept on ice for 1 h and centrifuged at 13,000 rpm for 2 min at 4°C to obtain supernatants that contained the mitochondrial fraction. Equal protein loading was verified by Bradford assay using the Bio-Rad Protein Assay (Bio-Rad Laboratories, Inc., Hercules, CA).

### Western blot analysis

All protein fractions were incubated with 2× sodium dodecyl sulfate (SDS) sample buffer for 4 min at 95°C, separated on 12% SDS-polyacrylamide gels, and electrophoretically transferred onto polyvinylidene difluoride membranes (Immobilon-P Transfer Membrane, Millipore). Membranes were blocked for 1 h at room temperature with 5% non-fat dried milk in phosphate buffered saline (PBS) containing 0.05% Tween-20 (PBS-t). After blocking, the membranes were incubated with anti-CYP24A1 monoclonal antibody (diluted 1:1,000 or 1:2,000; M02, Abnova, Taipei, Taiwan), which specifically recognizes a region in the C-terminus of the CYP24A1 protein, anti-β-actin monoclonal antibody (1:5,000 dilution; Sigma, MO), anti-COX 4 antibody (1:3,000 dilution; 3E11, Cell Signaling) or anti-lamin β polyclonal antibody (1:1,000 dilution; C-20, sc-6216, Santa Cruz Biotechnology, Santa Cruz, CA), and horseradish peroxidase (HRP)-labeled goat anti-mouse IgG (1:3,000 dilution; Invitrogen), goat anti-rabbit IgG (1:5,000 dilution; Bio Rad Laboratories, Inc.) or rabbit anti-goat IgG (1:1,000 dilution; Bio Rad Laboratories, Inc.). Anti-CYP24A1 monoclonal antibody was incubated overnight at 4°C with CYP24A1 recombinant protein (Q01, Abnova) in 1% non-fat dried milk/PBS-t before use in peptide neutralization analyses. Signals were detected using an ECL western blotting system or ECL prime western blotting system (GE Healthcare, Buckinghamshire, UK) on BioMax MR Film (Kodak, Rochester, UA).

### Cell culture

OK-P cells were cultured in Dulbecco’s modified Eagle’s medium (DMEM, Sigma) at 37°C with 5% CO_2_. The growth medium was supplemented with 10% fetal bovine serum (FBS, Sigma), 100 units/ml penicillin, and 0.1 mg/ml streptomycin (Sigma). Dexamethasone (DEX, Sigma), 1,25(OH)_2_D_3_ (Solvay Pharmaceuticals, GA), Actinomycin D (Act. D1 C1, Boehringer Mannheim, Mannheim, Germany) and RU 486 (Sigma) were used as reagents. OK-P cells grown to the confluence were treated with each reagent at different concentrations and times.

### Statistical analysis

Data are expressed as means ± SEM. Statcel2 (The Publishe OMS Ltd., Saitama, Japan) was used for intergroup significance difference tests. Date for two independent groups were analyzed by Student’s *t* test, Welch’s *t* test, or Mann-Whitney’s *U* test. Date for more than three independent groups were analyzed by one-way ANOVA with a post-hoc test of Tukey-Kramer test. *P*<0.05 was considered significant.

## Results

### Characteristics of diabetes in STZ rats

STZ rats showed significantly lower plasma insulin levels on Day 9 after STZ injection compared with control animals (Table 2). Furthermore, STZ rats had low body weight and high levels of food intake volume, plasma glucose, plasma corticosterone and 24 h urine volume (5 ml/24 h vs 213 ml/24 h for control and STZ rats, respectively), as previously reported.^(18,19)^ Plasma BUN levels were higher in STZ rats than the control. On the other hand, plasma creatinine levels were similar between control and STZ rats.

### Alterations in vitamin D-associated factors in STZ rats

**Fig. 1:**
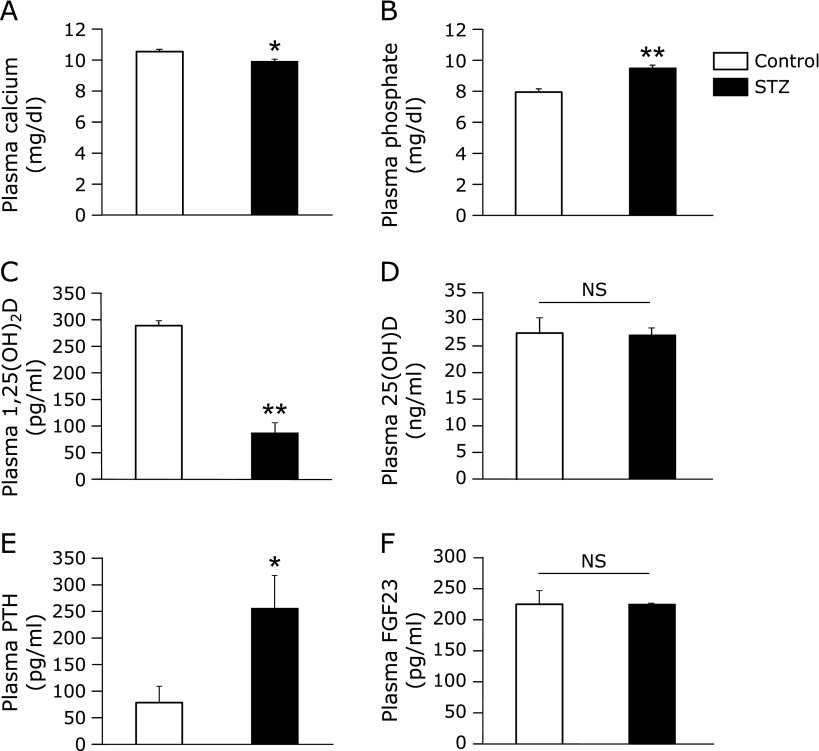
Plasma levels of calcium, phosphate and vitamin D-associated factors in STZ rats. Levels of plasma (A) calcium, (B) phosphate, (C) 1,25(OH)_2_D, (D) 25(OH)D, (E) PTH and (F) FGF23 were measured in plasma collected from the abdominal aorta of control and STZ rats. Values are expressed as means ± SEM. *p<0.05, **p<0.01 vs control. NS, not significant.

**Fig. 2:**
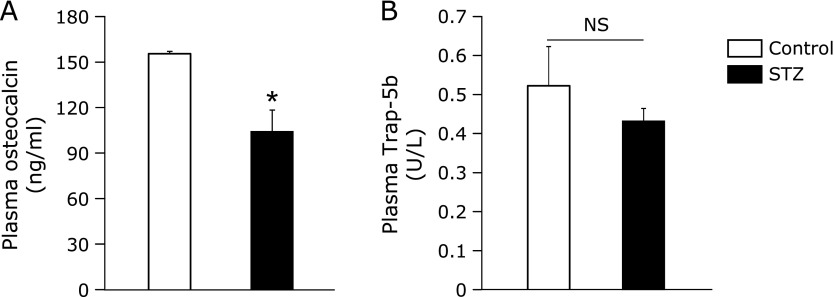
Osteocalcin and Trap-5b levels in plasma from STZ rats. Levels of plasma (A) osteocalcin and (B) Trap-5b were measured in plasma collected from the abdominal aorta of control and STZ rats. Values are expressed as means ± SEM. *p<0.05 vs control. NS, not significant.

Plasma Ca levels were lower, and plasma Pi levels were slightly higher in STZ rats compared to the control (Fig. 1A and B). Plasma 1,25(OH)_2_D levels in STZ rats were 30% that of the control, although there was no difference in plasma 25(OH)D levels between control and STZ rats (Fig. 1C and D). Plasma PTH levels were high in STZ rats, but there was no difference in plasma FGF23 levels (Fig. 1E and F). Furthermore, based on the observed changes in Ca and Pi homeostasis, we examined bone metabolism by measuring levels of the bone formation marker osteocalcin and the bone resorption marker Trap-5b. Plasma osteocalcin levels were lower in STZ rats than the control, but plasma Trap-5b levels were similar (Fig. 2).

### Expression levels of renal *Cyp27b1*, *Cyp24a1*, *Trpv5* and duodenal *Trpv6* mRNA in STZ rats

**Fig. 3:**
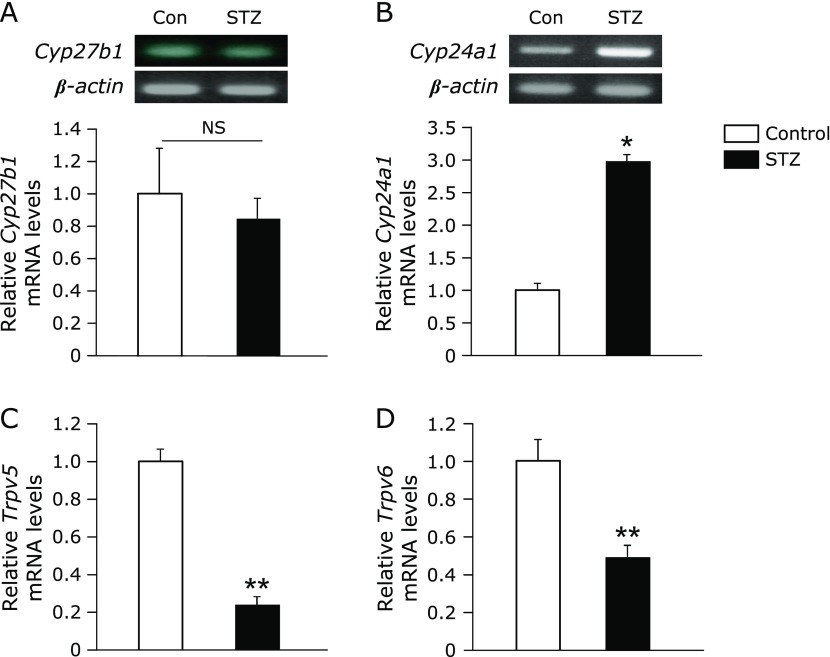
Expression levels of renal *Cyp27b1*, *Cyp24a1*, *Trpv5* and duodenal *Trpv6* mRNA in STZ rats. Relative mRNA levels for (A) *Cyp27b1*, (B) *Cyp24a1*, (C) *Trpv5* in the kidney and (D) *Trpv6* in the duodenum of control and STZ rats as measured by RT-PCR and/or quantitative real-time PCR. (B) Analysis performed using primer *Cyp24a1* Ex2-Ex4 (Table 1). Values are expressed as means ± SEM. *p<0.05, **p<0.01 vs control.

To determine the cause of the low 1,25(OH)_2_D levels in STZ rats, we examined mRNA expression level of genes associated with vitamin D metabolism. For the vitamin D synthetic enzyme *Cyp27b1*, mRNA (Fig. 3A) and protein (data not shown) expression levels in kidney were not different between control and STZ rats. Meanwhile, quantitative real-time PCR using the primer *Cyp24a1* Ex2-Ex4 (Table 1) showed that mRNA levels of the vitamin D catabolic enzyme *Cyp24a1* in kidney were 3-fold higher in STZ rats compared with the control (Fig. 3B). Based on the results showing that plasma Ca levels were low in STZ rats, we measured mRNA levels of *Trpv5* and *Trpv6* which act as calcium channels in the kidney and the duodenum, respectively. The amount of mRNA for both renal *Trpv5* and duodenal *Trpv6* was decreased in STZ rats compared to the control (Fig. 3C and D).

### High Cyp24a1 protein expression in STZ rat kidneys

**Fig. 4:**
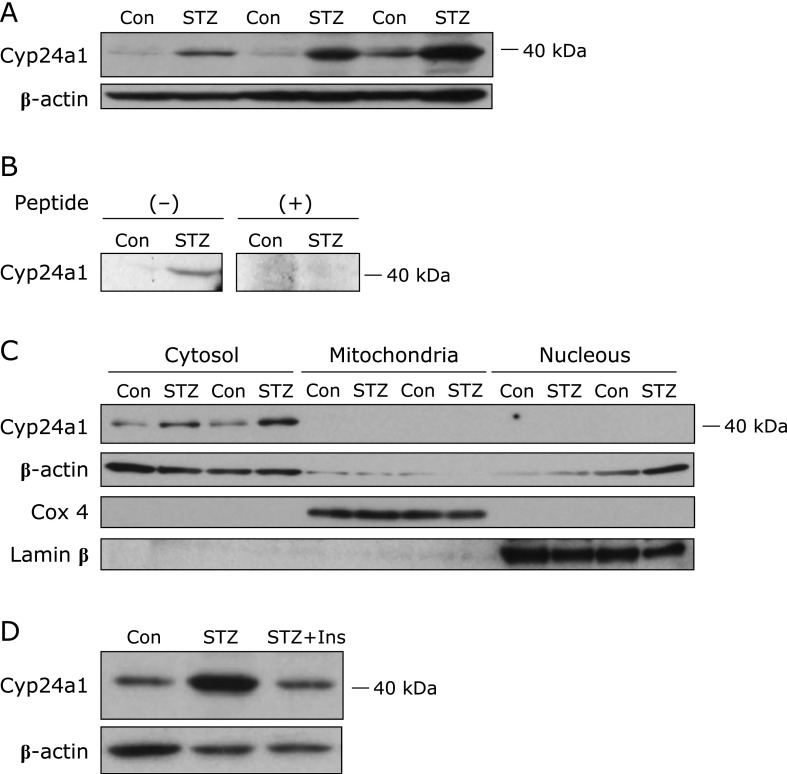
Renal Cyp24a1 protein expression in STZ rats, and effect of insulin treatment on Cyp24a1 expression. Cyp24a1 protein expression in the kidney from control and STZ rats was determined by western blot analysis using anti-CYP24A1 antibody. (A) Whole cell protein (30 µg) was used for the analysis and for (B) peptide neutralization tests, which were used to verify an anti-CYP24A1 antibody specificity. (C) Levels of Cyp24a1 protein expression in kidney cells fractionated into cytosolic (10 µg), mitochondrial (10 µg) and nuclear fractions (5 µg). (D) Whole cell protein (15 µg) was used for the analysis. STZ + Insulin rats were treated subcutaneously with 2 U insulin twice daily from Day 4 to Day 9.

Western blot analysis using an anti-CYP24A1 monoclonal antibody that specifically recognizes a region in the CYP24A1 protein C-terminus showed a strong signal at about 40 kDa in STZ rat kidneys (Fig. 4A). We confirmed that this signal was specific to the CYP24A1-antibody by peptide neutralization analysis using CYP24A1 recombinant protein (Fig. 4B). However, according to previous reports, the predicted size of full-length Cyp24a1 protein (WT-Cyp24a1) is 50–55 kDa.^(8,20)^ Interestingly, we observed the signal at about 40 kDa only in the cytosolic fraction, and not the mitochondrial and nuclear fractions (Fig. 4C). The signal at about 40 kDa was also found in STZ rat kidneys at Day 29 after STZ injection, and it was much stronger than the control (date not shown). The intensity of the the signal at about 40 kDa in STZ rats was decreased by insulin treatment (Fig. 4D). These results suggest that the signal at about 40 kDa might not be WT-Cyp24a1, but instead, be Cyp24a1-SV.

### Renal *Cyp24a1-SV* mRNA expression and insulin treatment effect on STZ rats

**Fig. 5:**
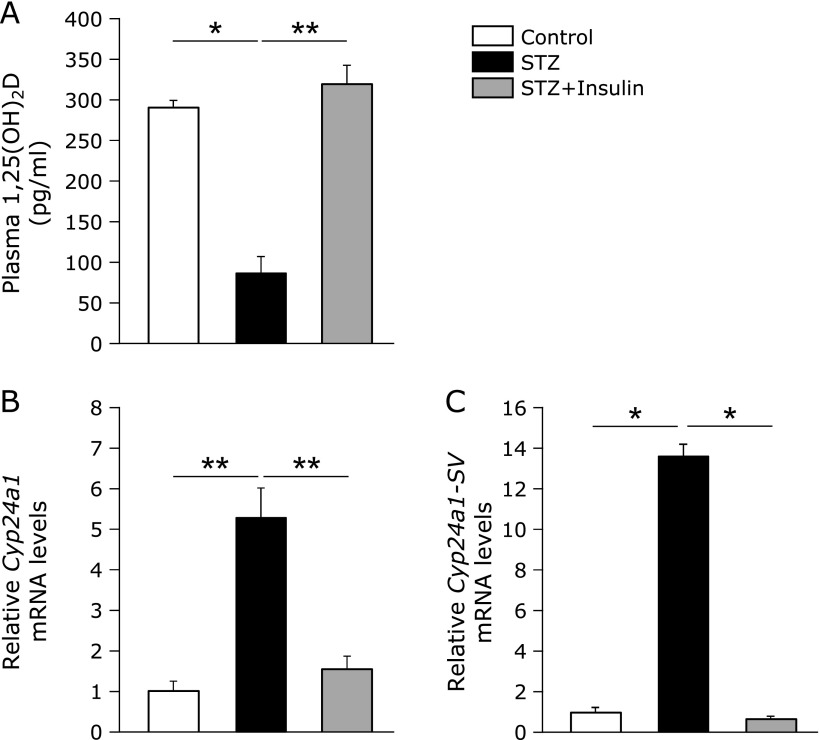
Plasma 1,25(OH)_2_D levels and renal *Cyp24a1* and *Cyp24a1-SV* mRNA expression in STZ rats. (A) Plasma 1,25(OH)_2_D levels, (B) renal *Cyp24a1* and (C) renal *Cyp24a1-SV* mRNA levels were measured in control and STZ rats. STZ + Insulin rats were treated subcutaneously with 2 U insulin twice daily from Day 4 to Day 9. Relative *Cyp24a1* and *Cyp24a1-SV* mRNA levels in kidney were measured by quantitative real-time PCR. The analysis were performed using (B) primer *Cyp24a1* Ex2-Ex4 and (C) primer *Cyp24a1-SV* Int2-Ex4 (Table 1). Values are expressed as means ± SEM. *p<0.05, **p<0.01. NS, not significant.

To assess renal *Cyp24a1-SV* mRNA expression, we designed a specific primer set that targets intron 2 and exon 4 of *Cyp24a1* (Table 1). *Cyp24a1-SV* mRNA expression was detected in the kidney of rats, and the levels were increased in STZ rats relative to control rats (Fig. 5C). In addition, insulin treatment of STZ rats ameliorated the low plasma 1,25(OH)_2_D levels (Fig. 5A) and decreased the high mRNA expression levels of both *WT-Cyp24a1* and *Cyp24a1-SV* observed in STZ rats (Fig. 5B and C). Furthermore, on Day 29 after STZ treatment STZ rats showed markedly lower plasma 1,25(OH)_2_D levels than on Day 9 (data not shown). Plasma 25(OH)D levels did not differ between STZ rats on Day 9 and on Day 29 (data not shown). Renal *WT-Cyp24a1* and *Cyp24a1-SV* mRNA levels in STZ rats on Day 29 were significantly increased, and the mRNA levels were much higher than STZ rats on Day 9 (data not shown).

### Effect of DEX on endogenous *Cyp24a1* expression in OK-P cells

**Fig. 6:**
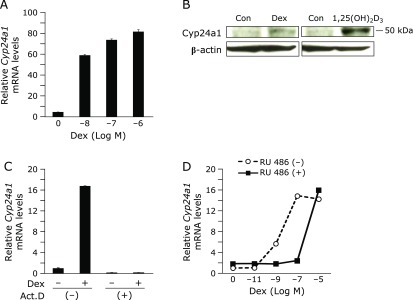
Effect of DEX on Cyp24a1 expression in OK-P cells. (A) OK-P cells were incubated with ethanol (EtOH) or 10^−8^, 10^−7^, 10^−6^ M of DEX for 24 h. (B) OK-P cells were incubated with EtOH, 10^−7^ DEX or 10^−8^ M 1,25(OH)_2_D_3_ for 22 h. Cyp24a1 protein expression in whole cell protein (30 µg) was determined by western blot analysis using anti-CYP24A1 antibody. (C) OK-P cells were incubated with or without 1 µg/ml Act. D for 30 min, and then the cells were incubated with EtOH or 10^−7^ M DEX. (D) OK-P cells were incubated with (closed squares) or without (open circles) 10^−6^ M RU 486 and EtOH or 10^−11^, 10^−9^, 10^−7^, 10^−5^ M DEX for 24 h. (A, C, D) Relative *Cyp24a1* mRNA levels were measured by quantitative real-time PCR.

Finally, we determined whether glucocorticoid treatment enhances renal *Cyp24a1* expression, because plasma corticosterone levels were higher in STZ rats than the control (Table 2), and these high levels could be decreased by insulin treatment (data not shown). DEX treatment dose-dependently enhanced endogenous *Cyp24a1* expression at both the mRNA and protein level in OK-P cells (Fig. 6A and B). Furthermore, the high endogenous *Cyp24a1* mRNA expression induced by DEX treatment was reduced by Act. D, which inhibits transcription (Fig. 6C), and by the glucocorticoid receptor inhibitor RU486 (Fig. 6D).

## Discussion

In this study, we investigated the relationship between change in Cyp24a1 expression change and abnormalities in vitamin D metabolism in rats with STZ-induced diabetes. We found that STZ rats showed typical features of type 1 diabetes, manifested as low insulin levels and high glucose levels in the plasma (Table 2). The high plasma BUN levels in STZ rats were likely due to dehydration during diabetes. Plasma creatinine levels in STZ rats were similar to control rats. As shown by Zhang *et al.*,^(21)^ the significant elevations in 24 h urine protein levels in diabetic nephropathy could be observed 8 weeks after STZ injection into rats, which is consistent with our finding that renal function of STZ rats at Day 9 after STZ injection was not severely impaired.

Plasma 1,25(OH)_2_D levels were significantly lower in STZ rats than the control (Fig. 1C). We also observed low levels of plasma Ca and the bone formation marker osteocalcin (Fig. 1A and 2A), as well as high levels of plasma PTH (Fig. 1E) in STZ rats. Meanwhile, mRNA expression levels of the calcium channels *Trpv5* and *Trpv6* in the kidney and the duodenum, respectively, were decreased in STZ rats (Fig. 3C and D). These results suggested that the low plasma 1,25(OH)_2_D levels could lead to the low plasma Ca levels due to the reducing of *Trpv5* and *Trpv6* inductions by 1,25(OH)_2_D. The Ca imbalance could then subsequently increase plasma PTH levels and inhibit bone formation.

To determine the basis of low plasma 1,25(OH)_2_D levels in STZ rats, we examined mRNA expression levels of *Cyp27b1* and *Cyp24a1*, which regulate vitamin D synthesis and catabolism, respectively, in the kidney of STZ and control rats (Fig. 3A and B). Although *Cyp27b1* mRNA expression was similar between STZ and control rats, *Cyp24a1* mRNA expression was significantly higher in STZ rats than the control. These results suggested that the low plasma 1,25(OH)_2_D levels in STZ rats were mainly due to an increase in renal *Cyp24a1* expression that affected vitamin D catabolism, rather than changes in *Cyp27b1* expression that would affect vitamin D synthesis.

However, in western blotting analysis of kidney tissues from STZ rats using an anti-CYP24A1 antibody that specifically recognizes a region in the C-terminus of the CYP24A1 protein (Fig. 4), we observed a strong signal at about 40 kDa, which is lower than the 50–55 kDa expected for Cyp24a1. Interestingly, Ren *et al.*^(8)^ reported the presence of a splicing variant, *CYP24A1-SV*, in the *CYP24A1* gene of humans and chicks. They found that *CYP24A1-SV* was spliced from intron 2 and lacked exons 1 and 2, to yield a 36 kDa CYP24A1-SV protein that is smaller than the 50–55 kDa WT-CYP24A1 protein. Based on these earlier observations and our analysis of *Cyp24a1-SV* mRNA expression (Fig. 5C), the signal at about 40 kDa on Western blotting analysis likely corresponds to the Cyp24a1-SV protein. The activity of mitochondrial cytochrome P450 enzymes, including CYP24A1, are dependent on the mitochondrial NADPH-adrenodoxin-reductase electron transport system.^(7)^ However, CYP24A1-SV protein lacks the N-terminal mitochondrial targeting domain encoded by exon 1 of *CYP24A1* gene.^(8,22)^ Therefore, CYP24A1-SV protein may remain in cytosol and be functionally inactive. Indeed, the signal at about 40 kDa was seen only in the cytosolic fraction, and not in the mitochondrial and nuclear fractions (Fig. 4C). Previous reports suggested that CYP24A1-SV could bind various substrates such as 25(OH)D_3_ and 1,25(OH)_2_D_3_ since CYP24A1-SV likely retains the substrate-binding domains encoded by exons 8 and 9 of the *CYP24A1* gene.^(8,22)^ Ren *et al.*^(8)^ showed that 1,25(OH)_2_D synthesis in cells was suppressed by *CYP24A1-SV* overexpression, but was increased in the presence of an antisense *CYP24A1-SV*. Thus, CYP24A1-SV may inhibit the entry of 25(OH)D_3_ into mitochondria by binding 25(OH)D_3_ in the cytosol. The elevated renal *Cyp24a1-SV* expression could also be associated with the low plasma 1,25(OH)_2_D levels seen in STZ rats.

Although renal *Cyp24a1* mRNA expression was increased in STZ rats (Fig. 3B and 5B), we did not observe WT-Cyp24a1 protein expression clearly. We guessed one possible cause of this result is that the anti-CYP24A1 antibody used here may recognize a version of the CYP24A1 protein that is smaller than WT-CYP24A1. Based on the effect of insulin treatment on STZ rats in which decreases in plasma 1,25(OH)_2_D levels and increases in *WT-Cyp24a1* and *Cyp24a1-SV* expression were inhibited (Fig. 5), insulin treatment likely can regulate not only blood glucose levels, but also 1,25(OH)_2_D blood levels through the inhibition of renal *Cyp24a1* gene expression.

Some studies reported low plasma PTH levels in rats with STZ-induced diabetes in a long experimental period of 7 weeks.^(23,24)^ Meanwhile, high plasma PTH levels in STZ-induced diabetes were also observed in previous studies that also included a 9 day experimental period.^(25)^ We believed that blood PTH levels may differ between the acute and chronic phases of diabetes.

There are some reports that diabetes is associated with FGF23 levels. In some studies of diabetes in humans, higher serum levels of FGF23 were seen in diabetes patients compared to healthy individuals.^(26,27)^ Bär *et al.*^(28)^ observed that serum FGF23 levels were significantly increased in mice with STZ-induced diabetes compared with control mice in a short experimental period of 10 days, and that these levels returned to control levels following insulin therapy. Similar results were also observed in a long experimental period of 9 weeks.^(29)^ FGF23 suppresses 1,25(OH)_2_D_3_ production by inhibiting *CYP27B1* expression and enhancing *CYP24A1* expression in the kidney,^(30)^ whereas *FGF23* expression is regulated by blood Pi level, PTH, 1,25(OH)_2_D_3_, and insulin.^(28,30)^ Bär *et al.*^(28)^ reported that insulin suppresses FGF23 production. Although the levels of all of these factors were altered in STZ rats, interestingly, there was no difference in plasma FGF23 levels between control and STZ rats (Fig. 1F). In addition, renal *FGF23* mRNA expression was not affected in STZ rats (data not shown). Although we can not say definite as serum insulin levels were not measured in Bär *et al.* study, the difference between our finding and that of Bär *et al.* could be attributed to the decrease level in blood insulin levels induced by STZ injection. In the Bär *et al.* study, DBA/2N mice were injected ip with 40 mg/kg B.W. STZ daily for 5 days, whereas we injected ip 65 mg/kg B.W. STZ into rats only once.

Based on our finding that plasma PTH levels increased and plasma FGF23 levels remained unchanged in STZ rats (Fig. 1E and F), we considered whether other factors besides PTH and FGF23 could enhance renal *Cyp24a1* expression. Injection of DEX was reported to enhance renal *Cyp24a1* expression both *in vivo* and *in vitro*.^(31–33)^ Our analysis of OK-P cells (Fig. 6) suggested that endogenous *Cyp24a1* expression could be transcriptionally enhanced through glucocorticoid receptors. Several studies reported that the glucocorticoid receptor inhibitor RU486 mitigated cognitive dysfunction during diabetes^(34)^ and the hypoglycemic effect.^(35,36)^ Furthermore, we found that RU486 treatment of OK-P cells reduced increases in endogenous *Cyp24a1* mRNA expression induced by DEX treatment, suggesting that RU486 could counteract decreases in 1,25(OH)_2_D_3_ levels during diabetes. Taken together, these findings suggest that RU486 could represent a new therapeutic agent for diabetes.

In conclusion, our study indicated that alterations in renal *Cyp24a1* expression rather than renal *Cyp27b1* expression may be the primary cause to decrease in plasma 1,25(OH)_2_D levels in rats with STZ-induced diabetes. We provide, to our knowledge, the first evidence demonstrating the presence of Cyp24a1-SV in kidneys of STZ rats. Our results suggest that high plasma corticosterone levels in diabetes may be one factor that induces transcriptional up-regulation of *Cyp24a1* gene.

## Figures and Tables

**Table 1:** The primer sequences for PCR amplification

Gene name	Forward sequence (5' to 3')	Reverse sequence (5' to 3')	Gene accession No.
Rat *Cyp24a1* Ex2-Ex4	GCAGAGTACCACAAGAAGTATGGC	AAGGACCACTTGTTCAGCTCAC	XM_006235672
Rat *Cyp24a1-SV* Int2-Ex4	TTGCGAGGCTCTAAGCACAGCC	AAGGACCACTTGTTCAGCTCAC	NC_005102.4
Rat *Cyp27b1*	CAGTTTCGGGAACCCAACTC	TGCAACTTCGTTTGCCAAAG	NM_053763
Rat *Trpv5*	CAAGAAGAAAGAGGCTCGTCA	GCAAAAGCAAAATAGGTTAGG	XM_006236376
Rat *Trpv6*	ACCAGAATGTGAACTTGGTCC	AAAATCGAGTGACCCCCAACC	XM_006236375
Rat *β-actin*	CTAAGGCCAACCGTGAAAAGA	TGGTACGACCAGAGGCATACA	XM_006248885
Opossum *Cyp24a1*	TCAAGCCCTGGAAAGCCTATCG	GAAGTCTGCCAAGACCTCATTGATTTT	XM_001378239
Opossum *β-actin*	CTGACCCTGAAGTACCCCATTGAACA	CTGGGGTGTTGAAGGTCTCAAACATG	XM_001362951

**Table 2:** Characteristics of diabetes in STZ rats

	Control	STZ
Body weight (g)	191.09 ± 4.37	143.28 ± 5.03**
Food intake (g/day)	18.66 ± 0.63	24.89 ± 0.20**
Plasma glucose (mg/dl)	117.2 ± 5.6	284 ± 39.1**
Plasma insulin (ng/ml)	1.92 ± 0.38	0.48 ± 0.12*
Plasma BUN (mg/dl)	7.58 ± 0.52	27.05 ± 3.86**
Plasma creatinine (mg/dl)	0.56 ± 0.06	0.73 ± 0.07
Plasma corticosterone (ng/ml)	108.24 ± 33.68	483.8 ± 108.6*

Body weight and food intake volume were measured dialy. Body weight showed was the value measured on Day 9 after STZ injection. Food intake volume showed was the mean for 9 days. Plasma was collected from the abdominal aorta of control and STZ rats. Values are expressed as means ± SEM. *p<0.05, **p<0.01 vs control.
